# Progress and hotspot of diet or exercise therapy in the treatment of non-alcoholic fatty liver disease

**DOI:** 10.3389/fnut.2024.1326092

**Published:** 2024-04-02

**Authors:** Xinyu Shi, Yalan Xiong, Hualing Song, Fen Rong, Nan Tang, Leping Zhu, Shenyu Li, Jianying Wang, Lei Zhang, Shengfu You, Guang Ji, Baocheng Liu, Na Wu

**Affiliations:** ^1^School of Public Health, Shanghai Innovation Center of Traditional Chinese Medicine Health Service, Shanghai University of Traditional Chinese Medicine, Shanghai, China; ^2^Institute of Digestive Diseases, Longhua Hospital, Shanghai University of Traditional Chinese Medicine, Shanghai, China; ^3^State Key Laboratory of Integration and Innovation of Classic Formula and Modern Chinese Medicine, Shanghai University of Traditional Chinese Medicine, Shanghai, China

**Keywords:** VOSviewer, CiteSpace, diet therapy, exercise therapy, non-alcoholic fatty liver disease

## Abstract

**Introduction:**

The primary treatment for non-alcoholic fatty liver disease (NAFLD) is modifying lifestyle through dietary or exercise interventions. In recent decades, it has received increasing attention. However, the lack of bibliometric analysis has posed a challenge for researchers seeking to understand the overall trends in this field.

**Methods:**

As of February 3rd, 2024, 876 articles on treating NAFLD through diet or exercise therapy from 2013 to 2023 had been retrieved. Two software tools, VOSviewer and CiteSpace, were utilized to analyze the growth of publications, countries, institutions, authors, journals, citations, and keywords. Additionally, the keywords with strong citation burstiness were identified to determine the changes and future trends of research hotspots in this field.

**Results:**

China had the highest number of articles, followed by the United States and South Korea. Yonsei University and *Nutrient*s were the institutions and journals with the most significant contributions. Professor Younossi Zobair M, from the United States, is the most prolific author in this field. Through analyzing the keywords, three research hotspots were identified: research on the pathogenesis of NAFLD, research on the treatment modalities of NAFLD, and research on the risk factors and diagnosis methods of NAFLD. In recent years, the research emphasis in this field has changed, suggesting that future research will focus on two frontier keywords: “oxidative stress” and “aerobic capacity.”

**Conclusion:**

In the past eleven years, the attention in this field was still rising, and the authors, journals, countries and so on had formed a considerable cooperative relationship. There were also many highly influential and productive researchers in this field. It is speculated that new research will continue around “aerobic exercise” and “oxidative stress” in the future.

## 1 Introduction

Non-alcoholic fatty liver disease (NAFLD) is a metabolic stress liver injury disease with more than 5% steatosis and no signs of hepatocyte damage, closely related to insulin resistance and genetic susceptibility (1). NAFLD is a clinicopathological syndrome characterized by abnormal accumulation of hepatic triglycerides and steatosis of hepatic parenchymal cells. The spectrum of diseases includes simple fatty liver disease, non-alcoholic steatohepatitis (NASH), liver cirrhosis, and hepatocellular carcinoma, and these diseases can develop continuously in some individuals (2). With the development of the economy, diet structure and lifestyle are also changing constantly, which leads to a substantial increase in the prevalence of obesity, metabolic syndrome, and type 2 diabetes mellitus (T2DM). A recent study found that the global prevalence of nonalcoholic fatty liver disease increased by 50.4% in 2019 compared with 1990, from 25.26 to 38.00%. Among them, the highest prevalence rate is in Latin America (44.37%), followed by the Middle East and North Africa (36.53%), South Asia (33.83%), Southeast Asia (33.0%), North America (31.20%), East Asia (29.71%), Asia-Pacific (28.02%) and Western Europe (25.10%) (3). As a metabolic disease involving many systems, NAFLD has seriously affected the health of people all over the world.

Currently, the primary goal of treating NAFLD is to lose weight and improve insulin resistance (IR). Among them, the essential factors that lead to obesity, including high-calorie diet structure, lifestyle with less exercise and more sitting, and lack of exercise, are also risk factors for NAFLD. Dietary treatment belongs to the category of modern nutrition. It is an important intervention to treat NAFLD by adjusting the daily diet and recipe collocation, improving human function, and regulating diseases. In 1913, Casimir Fink et al. (4) suggested that food contains “life amines.” This “biogenic amine” was separated in 1926. By 1950, all the major vitamins had been divided and synthesized to prevent and treat diseases related to nutritional deficiency. At the same time, due to the different levels of development in other countries, the long-term concern for specific nutrients has affected the direction of scientific research and policy intervention. From 1950 to 1970, Keys et al. (5) suggested that improper fat intake was the leading cause of heart disease. Yudkin et al. (6) indicated that excessive sugar intake was the leading cause of coronary heart disease, hypertriglyceridemia, and other diseases. Dietary fat and sugar were the new concerns in this period. From 1970 to 1990, more attention was paid to diet-related chronic diseases, including obesity, T2DM, and other chronic diseases. Since 1990, many experiments have proved that the single nutrition theory is insufficient to explain the influence of diet on diseases (7), which makes the research direction turn to the complex biological effects between food and dietary pattern and dietary structure. Exercise therapy, an essential part of physical therapy, is a method to treat body dysfunction and regulate diseases through various exercises and is a critical intervention method for treating NAFLD. Patients’ habits and preferences mostly influence sports, and there is no requirement for a specific form of sports (8).

Bibliometrics first appeared at the beginning of the 20th century and became an independent discipline in 1969, and it was widely used in literature analysis (9). Bibliometric analysis realizes clear and comprehensive graphic results through modern computer technology, which is helpful for data interpretation and provides a quantitative method for analyzing known literature in a specific field (9). It is crucial that the literature dosimetry analysis can reveal the internal relations between much basic information. Therefore, the development of a field can be observed by bibliometric analysis (10). The databases selected in this study were from the Web of Science (WOS) core collection. As a high-quality digital literature resource database, WOS has a high recognition among researchers and is considered the most suitable high-quality database for bibliometric analysis. CiteSpace and VOSviewer were used to draw the knowledge map. Both of the two software are commonly used tools for visualizing literature information. They have advantages in drawing the knowledge map, and their combination can complement each other (11). CiteSpace is software for measuring the similarity of knowledge units based on the data standardization method of set theory. It runs in a JAVA environment and uses the similarity algorithm in the time dimension to observe the process and historical span of knowledge evolution and to identify the changes and future trends of research hotspots in this field (12). VOSviewer is a software based on probability theory for data standardization. It can be used to construct a visual bibliometric network. It is less complicated, and the network categories for mapping knowledge are rich and beautiful (13).

Quite a few scholars are committed to diet or exercise therapy research for NAFLD, and their treatment methods are constantly improving. However, as far as we know, there is no research topic of bibliometric analysis. This study will reveal the field’s current development process and frontier trends through bibliometric analysis and provide the latest inspiration and decision-making basis for researchers and clinicians.

## 2 Materials and methods

### 2.1 Data collection

In this paper, the core collection of WOS is selected to ensure the comprehensiveness and accuracy of the retrieved data, considering that dietary intervention belongs to the category of modern nutrition; therefore, the retrieval strategy finally determined in this paper is [TS = (nutrition) OR TS = (exercise or “physical activity”)] AND TS = (“non-alcoholic fatty liver disease” or “non-alcoholic fatty liver”). The period was from January 2013 to December 2023, and the retrieval deadline was February 3rd, 2024. The only article was selected as the type of article, and there was no restriction on the language. In addition, for the accuracy of the data finally used for analysis, non-article literature was manually excluded. 876 documents were obtained through the search.

### 2.2 Data analysis

All recorded and referenced documents are exported as plain text files for those retrieved from the WOS core database. The exported record is “download_*.txt” for subsequent analysis. Microsoft Excel is used to analyze the amount of literature published in different years. Using VOSviewer 1.6.18 software, based on the probability theory data standardization method, a visual atlas of publishing countries, institutions, journals, and cited documents is constructed, and the cooperative network is analyzed. The size of a node is related to its degree, frequency of occurrence, and connection strength. The deeper and thicker the connecting lines between nodes, the stronger the connection and cooperation between the two nodes. The color of nodes represents different clusters. Using CiteSpaceV 6.1.R 6 software, a similarity algorithm in the time dimension draws the timeline and keyword burst diagrams. The timeline graph is a cluster graph, which can reflect the historical development of each cluster containing keywords over a while. Citation explosion is a crucial index to identify emerging trends. Both are used to determine the changes and future directions of research hotspots in this field.

## 3 Results

### 3.1 Information analysis of the published quantity

**Figure 1:**
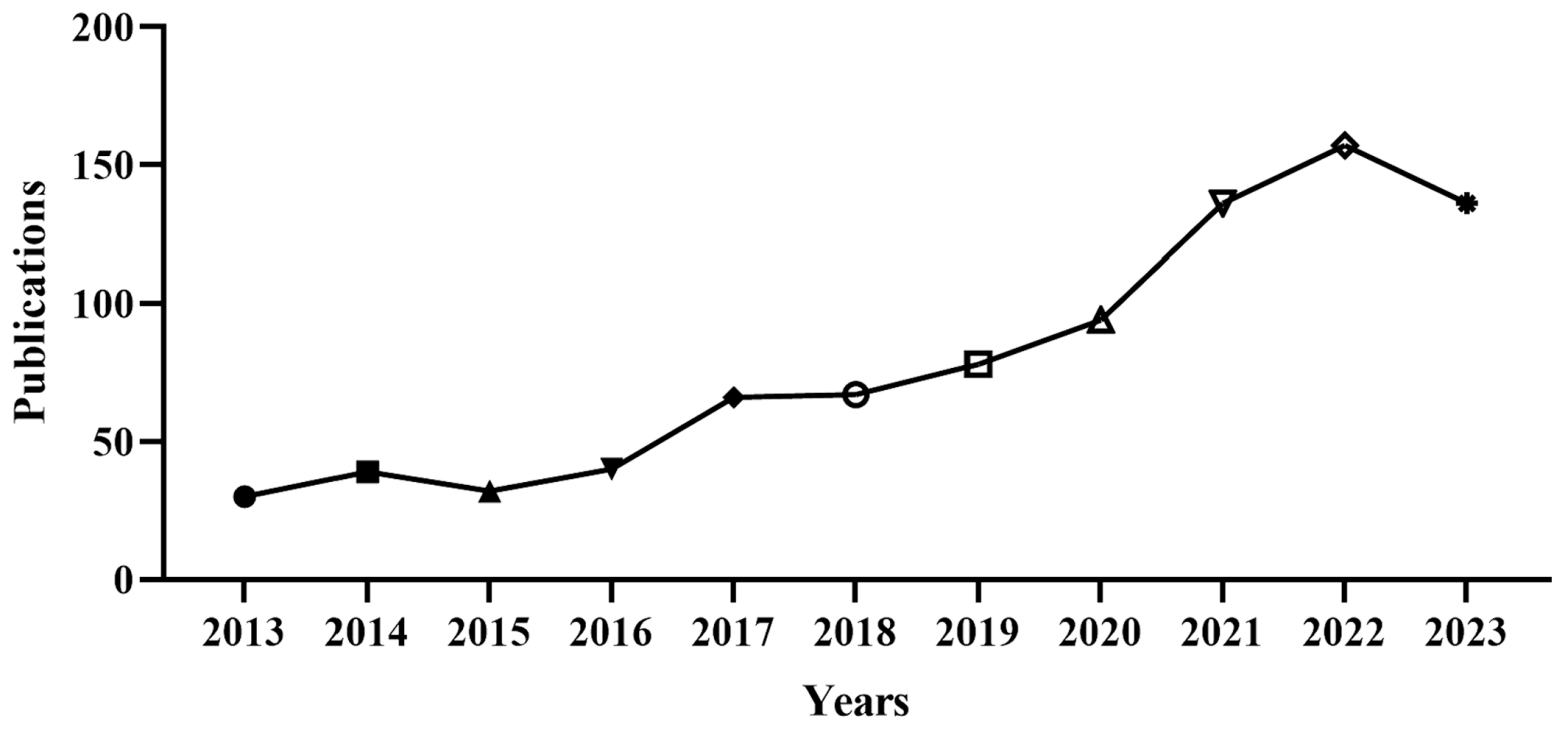
The annual distribution of publications. Annual distribution of diet or exercise therapy publications for non-alcoholic fatty liver disease from 2013 to 2023.

The 876 papers used in this study came from 5,631 authors of 1,058 institutions in 75 countries, published in 97 journals, and cited 13,286 references from 876 journals. This paper analyzes the information from articles published in the last decade. As shown in Figure 1, as of December 31st, 2023, there were 876 related research results on treating NAFLD with diet or exercise. Judging from the number of papers published each year, although there was a slight decline in 2023, the number of articles published in this field is on the rise, indicating that scholars have paid this research field more and more attention in recent years. Especially after 2020, the number of published articles increased significantly, and the number of published articles remained at more than 100 in 2021–2023. It was predicted that research achievements in diet or exercise therapy for NAFLD will continue to increase.

### 3.2 Information analysis of countries and periodicals

**Figure 2:**
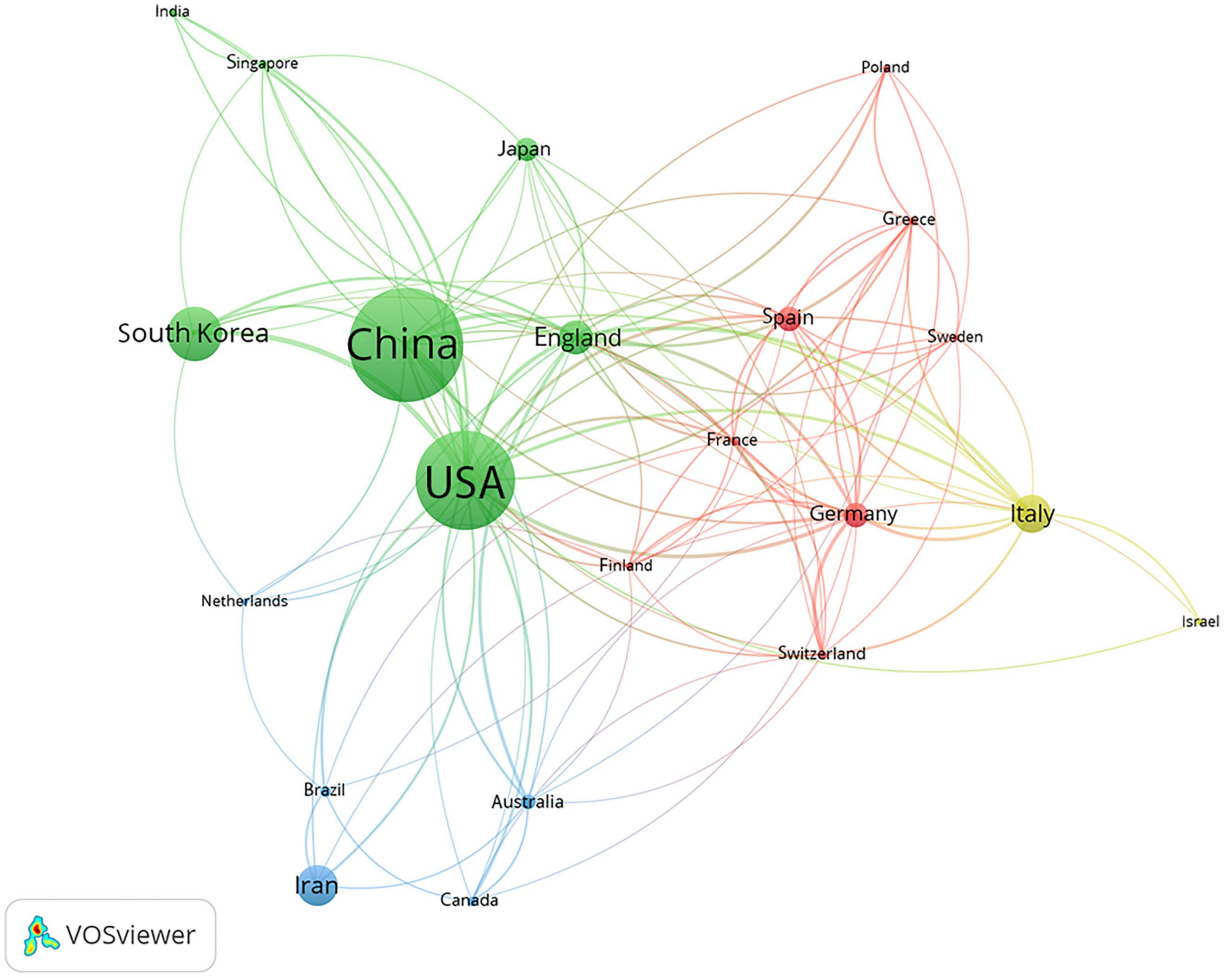
Analysis of national cooperation based on the VOSviewer visualization map. The node size is positively correlated with the frequency of node content, and the thickness of the connection line is positively correlated with the co-occurrence times of node content at both ends. Different color clustering reflects the cooperative relationship between countries.

China scholars have had the most fruitful research results in this field, with 214 papers published, accounting for 24.43% of the total number of papers published in this field, Followed by the United States, with a total of 188. These two countries published far more articles than other countries. In addition, South Korea, Italy, Iran, and other countries have also published several articles. From a centripetal point of view, the United States and England were the strongest, with 0.65 and 0.38, respectively, indicating that the two countries have conducted cooperative research with more countries, indicating that the United States and England were not only high-level and had many research results in the field of diet or exercise therapy for NAFLD, but also pay more attention to cooperation between countries than other countries (Table 1). In addition, among different continents, Asia published the most related articles, with a total of 516 articles, followed by Europe, with a total of 394 articles; in different regions, East Asia published the most related articles, with a total of 362 articles, accounting for 41.32% of the total number of articles (Table 2). Different colors represent different node groups that work closely together. The United States was at the center of the whole picture. It cooperated with many countries, which showed that the United States occupied a significant position in national scientific research cooperation and had made outstanding contributions. At the same time, the distribution of countries in this field was uneven, and the top effect was pronounced. Scholars from several countries wrote most papers. Green part: China, the United States, South Korea, Japan, England, Singapore, and India had close cooperation; Red part: Spain, Germany, France, Sweden, Greece, Switzerland, Finland, and Poland had close cooperation; Yellow part: Italy and Israel were closely cooperating; Blue region: Canada, Australia, Iran, Netherlands, and Brazil had close cooperation (Figure 2).

**Table 1:** Top 10 countries in terms of publications and centrality.

Rank	Documents	Centrality	Country	Rank	Centrality	Documents	Country
1	241	0.11	China	1	0.65	188	United States
2	188	0.65	United States	2	0.38	66	England
3	103	0.05	South Korea	3	0.18	4	South Korea
4	77	0.06	Iran	4	0.11	214	China
5	73	0.09	Italy	5	0.10	14	Poland
6	66	0.38	England	6	0.10	6	Belgium
7	47	0.03	Spain	7	0.10	2	Ghana
8	46	0.08	Germany	8	0.09	73	Italy
9	45	0.00	Japan	9	0.08	46	Germany
10	30	0.07	Australia	10	0.07	16	France

**Table 2:** Ranking of the number of articles published in different continents/regions.

Rank	Continents	Documents	Rank	Regions	Documents
1	Asia	516	1	East Asia	362
2	Europe	394	2	North America	213
3	North America	213	3	South Europe	152
4	South America	34	4	West Asia	116
5	Oceania	33	5	West Europe	116
6	Africa	27	6	Central Europe	91

The journals with more than 25 articles were *Nutrients*, *Frontiers in Nutrition*, and *Clinical Nutrition*, with 65 pieces, 27 articles, and 25 articles, respectively. By analyzing the citation of journals, it was found that the journal with the highest average number of sources was *Journal of Hepatology*, with a total of 20 articles, with an average number of citations of 149.05 times and the highest impact factor of 25.70, which showed that the articles published in this journal are of high quality and have attracted a lot of attention in the field of diet or exercise therapy for NAFLD (Table 3).

**Table 3:** Top 10 journals in terms of publications and centrality.

Rank	Source	Documents	Citations	Average citation/publication	IF(2023)
1	Nutrients	65	926	14.25	5.90
2	Frontiers in Nutrition	27	113	4.19	5.00
3	Clinical Nutrition	25	821	32.84	6.30
4	Liver International	22	635	28.86	6.70
5	Nutrition Metabolism and Cardiovascular Diseases	22	218	9.91	3.90
6	Scientific Reports	21	261	12.43	4.60
7	Journal of Hepatology	20	2,981	149.05	25.70
8	Journal of Clinical Medicine	18	117	6.50	3.90
9	BMJ Open	17	213	12.53	2.90
10	Frontiers in Endocrinology	16	183	11.44	5.20

### 3.3 Information analysis of authors and institutions

**Figure 3:**
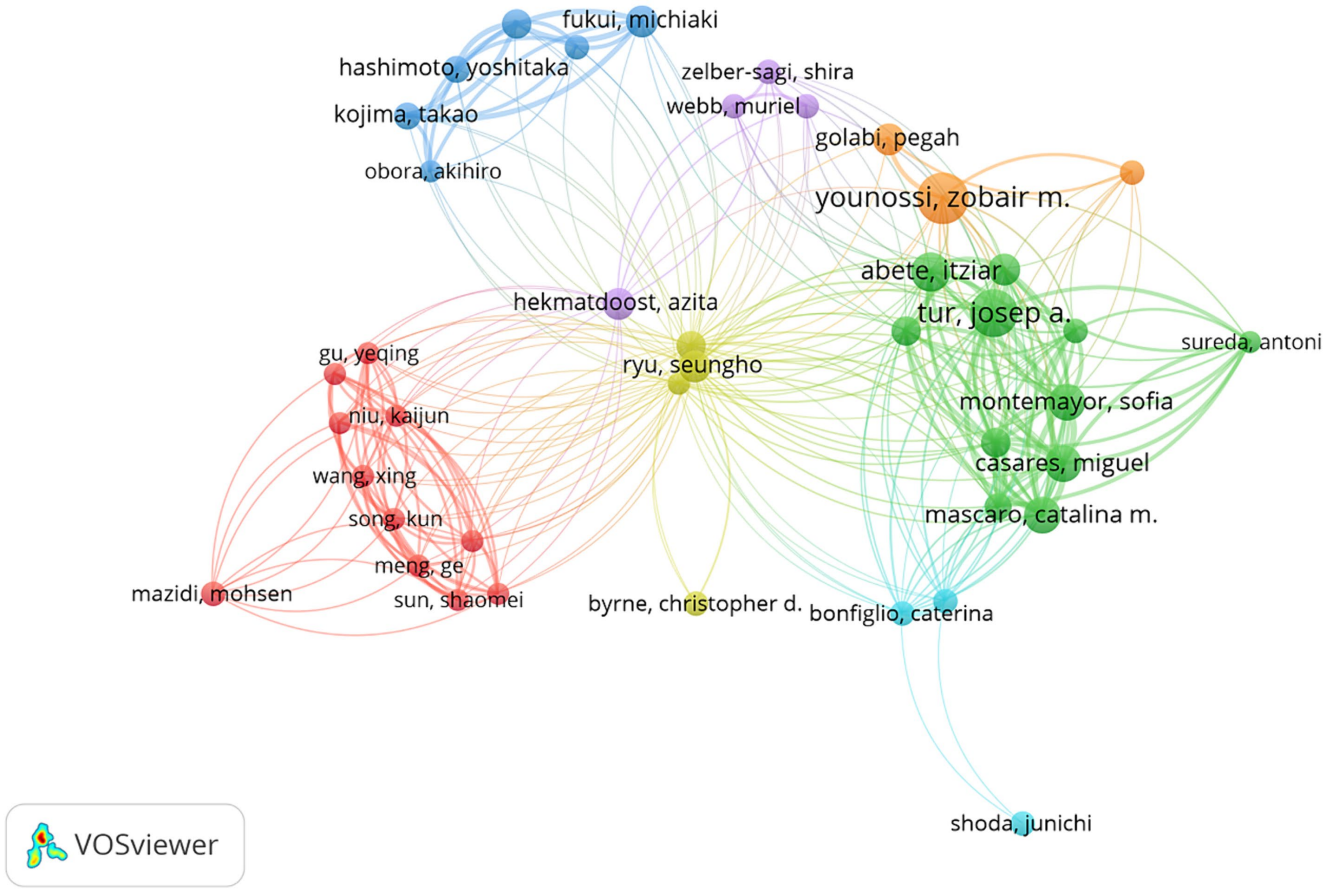
Analysis of author cooperation based on the VOSviewer visual map. The node size is positively correlated with the frequency of node content, and the thickness of the connection line is positively correlated with the co-occurrence times of node content at both ends; different color clustering reflects the cooperative relationship between authors.

The organizations of the top 10 high-productivity authors were mainly from Spain. Younossi Zobair M. from the Global NASH Committee of the United States published 19 articles from 2013 to 2023 and obtained 905 citations, each being cited 47.63 times. The second place was Tur, Josep A. from the University of the Balearic Islands in Spain, who published 17 papers and was awarded 132 times, with each article cited 7.76 times. Younossi Zobair M. was also the most frequently cited author, which shows that this author had a high influence in this field (Table 4). Different colors represent different clusters that work closely together. Overall, there was large-scale cooperation among researchers. Through research, we can find that authors in the same country cooperated more closely. Orange part: All authors were from Inova Health System in the United States; Green part: The authors were all from Spain; Dark blue part: The authors are all from Japan; Red part: The authors were all from universities in China (Figure 3).

**Table 4:** Top 10 the most productive authors distributed by publications and citations.

Rank	Author	Documents	Citations	Average citation/publication	Rank	Cited author	Citations
1	Younossi, Zobair M.	19	905	47.63	1	Younossi, Zobair M	557
2	Tur, Josep A.	17	132	7.76	2	Chalasani, N	243
3	Abete, Itziar	13	187	14.38	3	Targher, G	194
4	Casares, Miguel	12	87	7.25	4	Angulo, P	184
5	Mascaro, Catalina M.	12	87	7.25	5	Marchesini, G	161
6	Montemayor, Sofia	12	87	7.25	6	Zelber-sagi, S	153
7	Fukui, Michiaki	10	239	23.90	7	Bedogni, Giorgio	151
8	Golabi, Pegah	10	616	61.60	8	Musso, GAdams, La	128
9	Hekmatdoost, Azita	10	153	15.30	9	Lonardo, A.	115
10	Ryu, Seungho	10	546	54.60	10	Wong, Vws	105

**Figure 4:**
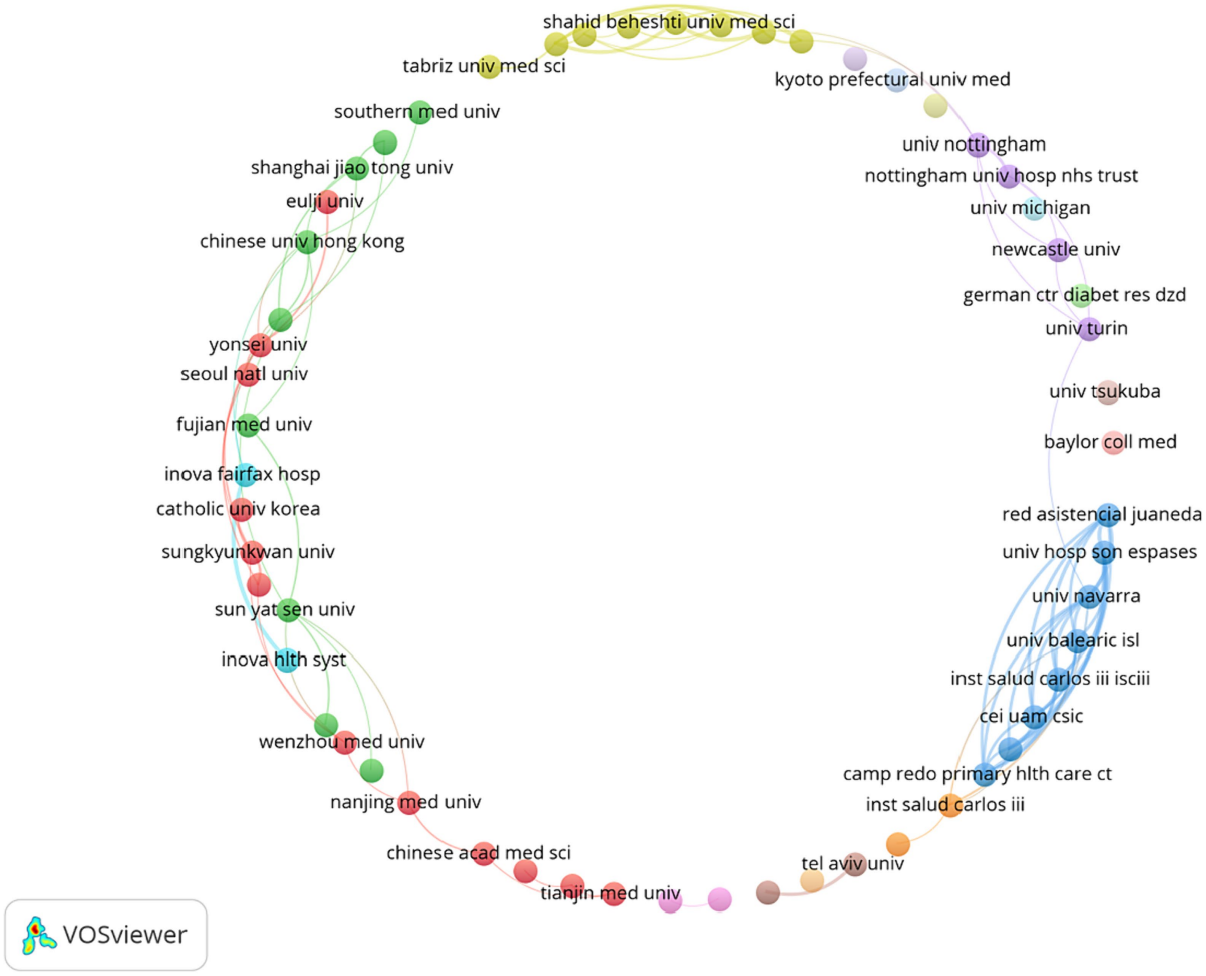
Analysis of institutional cooperation based on the VOSviewer visualization map. The node size is positively correlated with the frequency of node content, and the thickness of the connection line is positively correlated with the co-occurrence times of node content at both ends; different color clustering reflects the cooperative relationship between institutions.

According to the analysis in Table 5, the number of articles published by Yonsei University in South Korea was the largest, with 20 pieces, followed by Sungkyunkwan University in South Korea, with 19 articles, and the Public University of Navarre in Spain, with 18 reports. On the whole, most of the higher-ranked institutions were from South Korea, Spain, and Iran. These results showed that the institutions were leading in this field. From the perspective of inter-agency cooperation, Yonsei University, Iran University of Medical Sciences, and Sun Yat-sen University were highly concentrated, which showed that they pay more attention to cooperative research with other institutions than other institutions. As shown in Figure 4, the analysis diagram shows the distribution relationship of more than 10 mechanisms, and different colors indicate different node clusters, which are closely matched. At the same time, it can be seen that the blue part was the thickest part between nodes, indicating that these institutions have a very close cooperative relationship.

**Table 5:** Top 10 institutions with the most publications.

Rank	Documents	Centrality	Organization	Country
1	20	0.04	Yonsei University	South Korea
2	19	0.01	Sungkyunkwan University	South Korea
3	18	0.01	Public University of Navarre	Spain
4	18	0.01	Shahid Beheshti University of Medical Sciences	Iran
5	16	0.01	Inova Fairfax Hospit	United States
6	15	0.01	Tehran University of Medical Sciences	Iran
7	15	0.03	Iran University of Medical Sciences	Iran
8	13	0.00	Instituto de Salud Carlos III	Spain
9	13	0.01	University of the Balearic Islands	Spain
10	13	0.03	Sun Yat-sen University	China

### 3.4 Information analysis of cited documents

**Figure 5:**
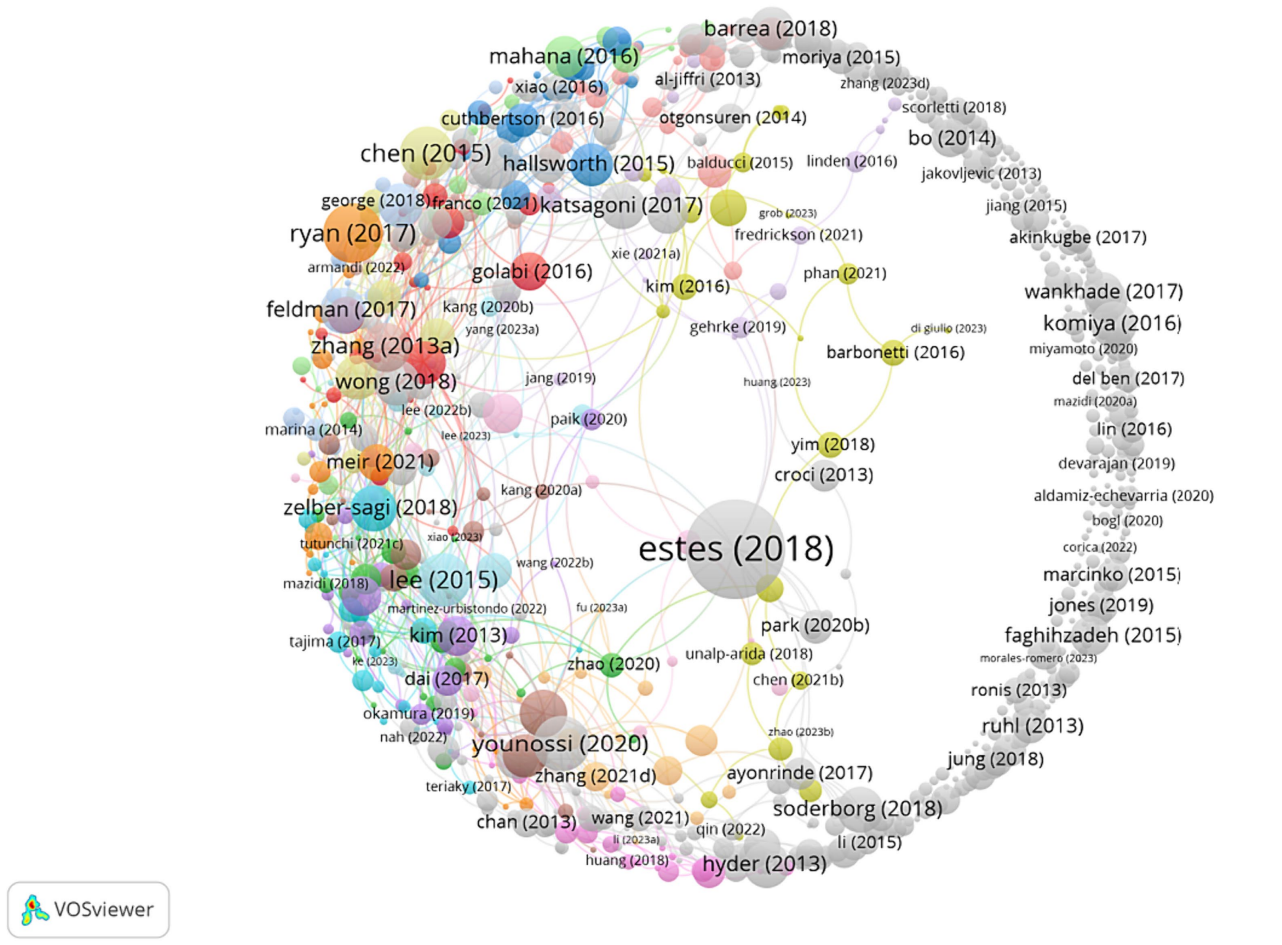
Analysis of co-cited literature based on the VOSviewer visualization map In network visualization, the size of nodes depends on the frequency of co-citation. The greater the citation frequency, the greater the contribution of the articles at both ends of the node to the future development of this field.

By presenting the top 10 cited articles in this field, this paper analyzes the highly cited articles of great significance. There were 31 articles cited more than 100 times, most of which were cross-sectional studies and randomized controlled studies, which increased the credibility of the research results (Table 6). The most frequently cited and core article was “Modeling NAFLD Disease Burden in China, France, Germany, Italy, Japan, Spain, United Kingdom, And United States for 2016–2030,” published in 2018 by Estes C. This paper used the Markov model to predict the disease burden of NAFLD using the currently available data. It concluded that the burden would continue to increase, showing the necessity and urgency of research on NAFLD’s treatment methods. This also means that as the primary treatment of NAFLD, diet and exercise therapy need to be further studied (Figure 5).

**Table 6:** Top 10 the most cited literature.

Rank	Count	Title	Author	Journal	Year	Volume	Issue	Page	DOI
1	963	Modeling NAFLD disease burden in China, France, Germany, Italy, Japan, Spain, United Kingdom, and United States for the period 2016–2030.	Estes, C	Journal of Hepatology	2018	69	4	896	10.1016/j.jhep.2018.05.036
2	267	Epidemiology of chronic liver diseases in the USA in the past three decades.	Younossi, ZM	Gut	2020	69	3	564	10.1136/gutjnl-2019-318813
3	234	Sarcopaenia is associated with NAFLD independently of obesity and insulin resistance: Nationwide surveys (KNHANES 2008–2011).	Lee, YH	Journal of Hepatology	2015	63	2	486	10.1016/j.jhep.2015.02.051
4	233	Resveratrol improves insulin resistance, glucose and lipid metabolism in patients with non-alcoholic fatty liver disease: A randomized controlled trial.	Chen, SH	Digestive and Liver Disease	2015	47	3	226	10.1016/j.dld.2014.11.015
5	216	Community-based lifestyle modification programme for non-alcoholic fatty liver disease: A randomized controlled trial.	Wong, VWS	Journal of Hepatology	2013	59	3	536	10.1016/j.jhep.2013.04.013
6	184	Irisin is inversely associated with intrahepatic triglyceride contents in obese adults.	Zhang, HJ	Journal of Hepatology	2013	59	3	557	10.1016/j.jhep.2013.04.030
7	179	Metabolic dysfunction-associated fatty liver disease is associated with increased all-cause mortality in the United States.	Kim, D	JOURNAL OF HEPATOLOGY	2021	75	6	1,284	10.1016/j.jhep.2021.07.035
8	167	Cadmium Exposure and Liver Disease among US Adults.	Hyder, O	Journal of Gastrointestinal Surgery	2013	17	7	1,265	10.1007/s11605-013-2210-9
9	165	High red and processed meat consumption is associated with non-alcoholic fatty liver disease and insulin resistance.	Zelber-Sagi, S	Journal of HepatOLOGY	2018	68	6	1,239	10.1016/j.jhep.2018.01.015
10	164	Beneficial effects of lifestyle intervention in non-obese patients with non-alcoholic fatty liver disease	Wong, VWS	Journal of Hepatology	2018	69	6	1,349	10.1016/j.jhep.2018.08.011

### 3.5 Keywords and hot spot analysis

Keywords reflect the core content of each article. By observing its historical development process and discovering unknown words, we can identify the changes and future trends of research hotspots in this field. By presenting the top 10 keywords in this field, this paper analyzes the overview of keywords in this field. By comparing the frequency and centrality of keywords, it is found that “non-alcoholic fatty liver,” “insulin resistance,” “metabolic syndrome,” “risk,” and “prevalence” have a higher frequency and occupy the central position of The relationship network. The high centrality of keywords such as “liver disease,” “population,” “weight lose,” and “cardiovascular disease” means that these keywords are closely related to other keywords. According to the frequency and centrality of keywords, we can judge the hot spots and frontiers in this field (Table 7).

**Table 7:** Top 10 keywords in terms of publications and centrality.

Rank	Count	Centrality	Keyword	Rank	Centrality	Count	Keyword
1	446	0.01	Non-alcoholic fatty liver	1	0.09	34	Liver disease
3	243	0.02	Risk	3	0.07	62	Weight lose
4	203	0.02	Metabolic syndrome	4	0.06	63	Cardiovascular disease
5	202	0.02	Prevalence	5	0.06	21	Skeletal muscle
6	187	0.02	Fatty liver	6	0.05	158	Obesity
7	176	0.04	Physical activity	7	0.05	125	Association
8	158	0.05	Obesity	8	0.05	112	Disease
9	142	0.02	Hepatic steatosis	9	0.05	87	Exercise
10	133	0.03	Steatohepatiti	10	0.05	66	Nash

**Figure 6:**
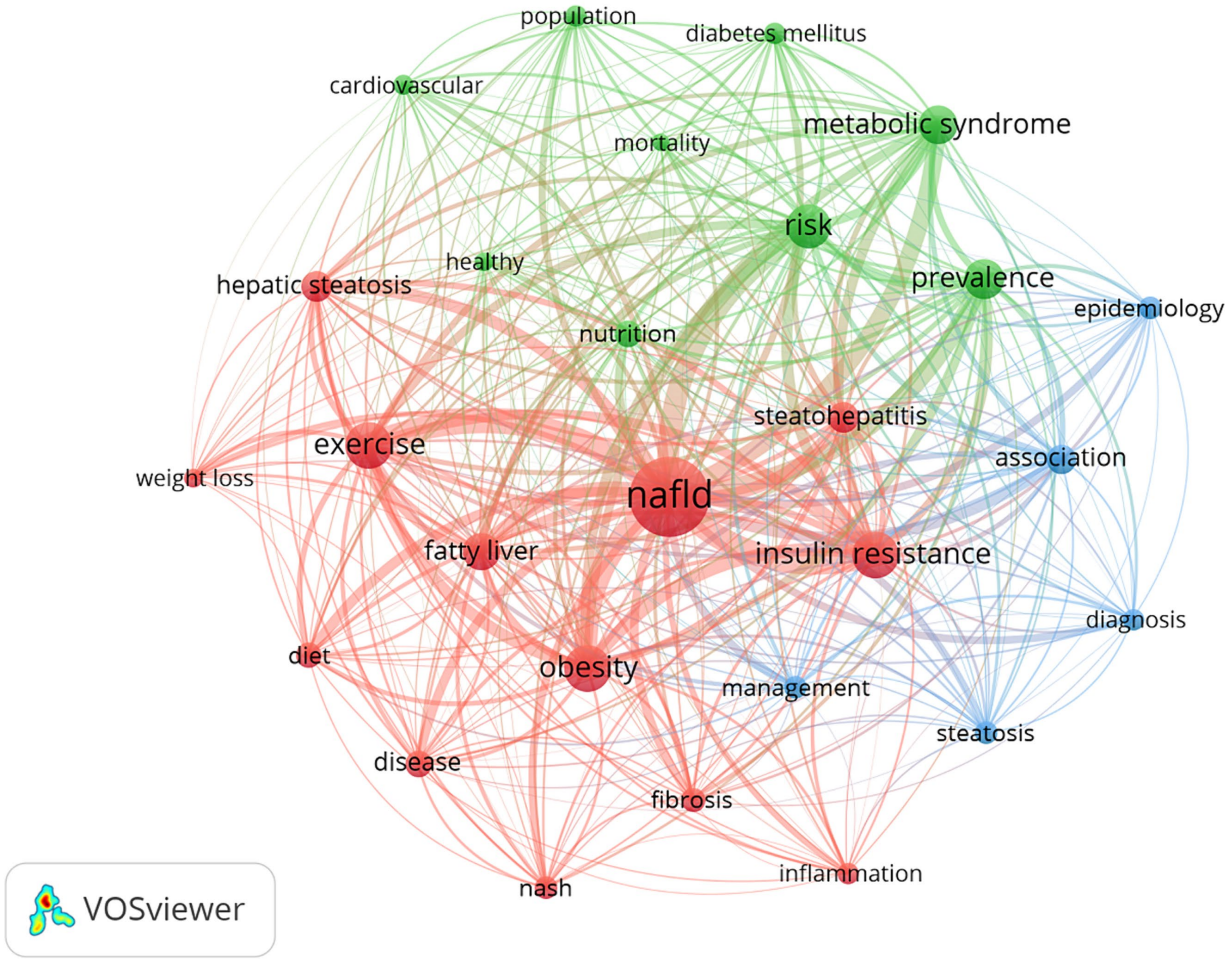
Analysis of keywords based on the VOSviewer visual map. The node size is positively correlated with the frequency of node content, and the thickness of the connection line is positively correlated with the co-occurrence times of node content at both ends; different color clustering reflects the cooperative relationship between keywords.

The keyword is the author’s refining and summarizing of the article’s content, which can reflect the core content of the article. In this paper, 55 times or more are set as high-frequency keywords, and VOSviewer is used to present the co-occurrence network of high-frequency keywords. The thickness of the node connection is positively related to the co-occurrence frequency of the nodes at both ends of the link. As shown in Figure 6 keywords mainly form three clusters, representing three main research directions in this field. The red part is primarily composed of “insulin resistance”, “obesity”, “inflammation”, “steatohepatitis”, “exercise”, “diet”, and so on, and is mainly involved in the pathogenesis and treatment of NAFLD. The green part is primarily composed of “risk”, “cardiovascular”, “diabetes mellitus”, “metabolic syndrome”, and, so on which are mainly related to the risk factors of NAFLD. The blue part is primarily composed of “steatosis”, “diagnosis”, “management”, and so on; this section mainly introduces the diagnosis and management methods in the development of NAFLD

**Figure 7:**
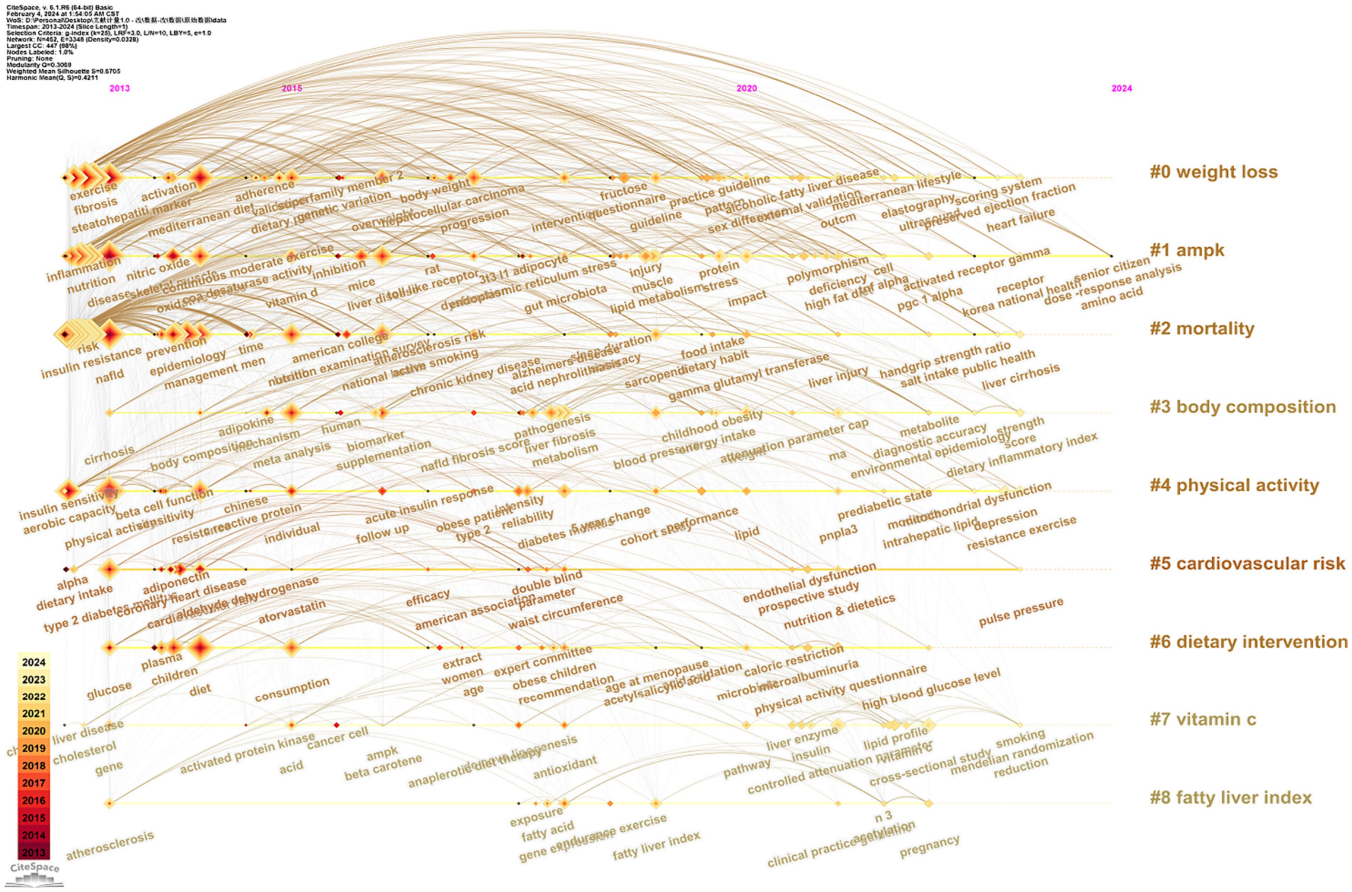
A timeline view of keywords. Each period corresponds to a longitudinal time axis, and the keywords on the time axis represent the first appearance of the keywords in this period; The node size represents the total frequency of this keyword for several years. The annual ring of node content, the color of each layer represents the year when the keyword reappears, and the thickness is the frequency of this year; Connecting means co-occurrence.

CiteSpace is a timeline graph used to draw keywords. The timeline of keywords is essentially a clustering diagram, which can observe the historical development of keywords in multiple clusters in 10 years. Wherein each period corresponds to a vertical time axis, and the keywords on the time axis represent the first appearance of the keywords in the period. The node’s size indicates the keyword’s total frequency in recent years. The annual ring of node content, the color of each layer suggests the year when the keyword reappears, and the thickness is the frequency of this year. Connecting means co-occurrence. We can get nine groups of high-frequency words representing nine main research directions in this field (Figure 7). The greater the number of clusters, the fewer keywords are included. They are “weight loss,” “ampk” (activated protein kinase), “mortality,” “body composition,” “physical activity,” “cardiovascular risk,” “dietary intervention,” “vitamin C,” and “fatty liver index.” Among them, “weight loss” and “ampk” were the rich categories of articles, and they were the most concerned research directions in this field, which not only have a large number of articles but also have new research content every year from 2013 to 2023. The last new research keywords, “dietary intervention” and “fatty liver index” appeared in 2022, and all the other clusters still have new research keywords.

**Figure 8:**
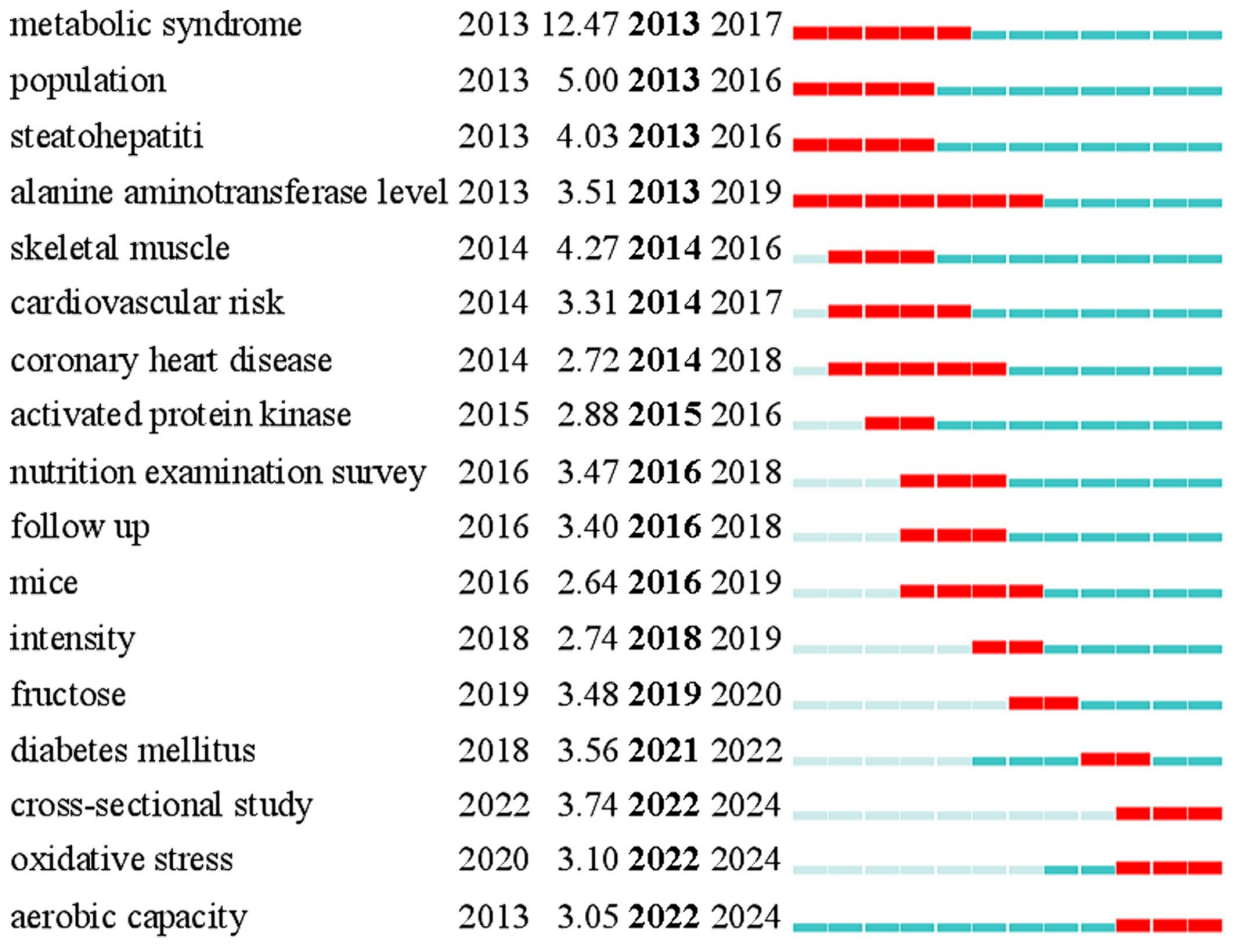
Top 17 keywords with the strongest citation bursts. The table shows the outbreak keywords in the field of diet or exercise therapy for non-alcoholic fatty liver disease in recent 10 years and ranks them according to the years when they appeared.

Keyword burst detection can identify the changes and future trends of research hotspots in this field. In the diagram, the blue line represents the timeline, and the red line represents the outbreak period. You can see the first appearance year, outbreak intensity, outbreak starts the year, and outbreak end year of many keywords in the picture. Through keyword burst detection, 17 outbreak keywords with the strongest references displayed in order of outbreak start year are obtained. From 2013 to 2019, more attention was paid to “metabolic syndrome,” “population,” “steatohepatitis,” “alanine aminotransferase level,” “skeletal muscle,” “cardiovascular risk,” “coronary heart disease,” “activated protein kinase,” “nutrition examination survey” and “follow up” (Figure 8). From 2019 to 2023, “mice,” “intensity,” “fructose,” and “diabetes mellitus” received more attention. From 2023 to 2024, “cross-sectional study,” “oxidative stress,” and “aerobic capacity” received more attention. We can infer this field’s research focus and frontier (Figure 8).

## 4 Discussion

In this study, VOSviewer 1.6.18 and CiteSpaceV 6.1.R 6 were used to analyze 876 articles on treating NAFLD by exercise or diet published in the core database of WOS from 2013 to 2023. The following main conclusions were drawn from time distribution, space–time distribution, the contribution of authors and articles, hot spots, and frontiers.

### 4.1 General description

The number of published articles increased from 2013 to 2023, especially after 2020. The number of published articles remained at more than 100 during 2021–2023, indicating a good development trend in this field, which may be attributed to the high prevalence rate of NAFLD caused by various factors, which attracted the attention of many scholars.

Researchers from China have made the most significant contribution to this field, accounting for 24.43% of the total number of papers published in this field, which may be attributed to the fact that the incidence of NAFLD in China has now exceeded the global incidence. At the same time, the United States and England have the highest centrality, which shows that researchers in these two countries paid more attention to academic cooperation. Among them, China ranked second in the number of papers published but fourth in the center, which may be why China started late. The number of papers published in East Asia was the largest, accounting for 41.32% of the total. In addition, the number of published articles was not directly related to the incidence of NAFLD in this area.

Among the top 10 organizations with the highest productivity, three organizations had a large number of publications from Iran. At the same time, the centrality of Yonsei University and Iran University of Medical Sciences was high, which showed that they have high collaboration ability and academic influence.

Younossi Zobair M., from the United States, was the author who has published the most articles in this field and was a member of the global Nash Committee. H index was as high as 103, which shows that he greatly influenced the research field. From the analysis of published papers, Younossi Zobair M. pays more attention to the diagnosis, treatment, pathological causes, and so on of NAFLD, among which he thinks that strenuous exercise is beneficial to adult patients with NAFLD, especially to adult NAFLD patients with metabolic abnormalities (14). The most frequently cited article was by Chris Estes from the US Centers for Disease Control and Prevention. His articles included epidemiological research and disease prediction model research of various liver diseases, which pointed out the direction for clinicians and essential research work.

The impact factor is a standard method to evaluate the quality of journals. According to our results, among the top 10 journals in terms of productivity, “Journal of Hepatology” had the highest impact factor of 25.70, and its 20 published articles have been cited 2,981 times, which indicated that the published articles were of high quality and have been recognized by peers in this field. “Journal of Hepatology” has become the top journal in this field. By analyzing the published literature, it was found that this journal mainly contained empirical research papers covering the whole area of medicine-gastrointestinal hepatology. Whether the influencing factors were high or low, journals were influential in promoting diet or exercise therapy to treat NAFLD.

### 4.2 Hot spots and frontiers

The keyword is the authors’ refining and summarizing of the content of the article, which can reflect the core content of the article. Based on keyword analysis, the main research direction of exercise or diet therapy in the field of NAFLD in the last 10 years was observed, that is, the research hotspot, including (1) Studies on the pathogenesis of NAFLD. IR caused by obesity and other factors is not only the strongest predictor of NAFLD (15), but also the critical factor leading to steatosis and abnormal accumulation of hepatocyte fat (16). At this time, abnormal liver fat accumulation will cause oxidative stress and lipid peroxidation, leading to mitochondrial dysfunction and inflammatory mediators and finally resulting in inflammatory necrosis and fibrosis of the liver (17). (2) Studies on the treatment of NAFLD. Many pieces of evidence showed that calorie restriction (18, 19), fat restriction (20, 21), carbohydrate reduction (22, 23), and appropriate vitamin supplementation (24, 25) in diet therapy, aerobic exercise (26, 27) resistance exercise (28, 29) and high-intensity interval training (30) in exercise therapy had good therapeutic effects. Moreover, clinical experiments have proved that combining diet and exercise may achieve better therapeutic efficacy than a single one (31, 32). (3) Studies on risk factors and diagnosis methods of NAFLD. In addition to obesity, dyslipidemia, T2DM, hypertension, and IR, genetic factors are also important risk factors for NAFLD (33–39), such as *PNPLA3* (40) and transmembrane 6 superfamily member 2 (*TM6SF2*) (41, 42) genes. In addition, intestinal flora disorder and the change of metabolites are important risk factors for NAFLD. Intestinal flora can lead to NAFLD by reducing intestinal barrier permeability and participating in immune inflammatory reactions. Targeted regulation of intestinal flora and its metabolites by changing diet and exercise has become a new treatment for NAFLD (43). To diagnose and distinguish the development stage of NAFLD and prevent or reverse the further progression of NAFLD in time, a large number of researchers have devoted themselves to developing non-invasive diagnostic methods (44), such as the serum inflammatory markers, apolipoprotein A-I (APAI1) (45).

Keyword burst detection can identify the change in research hotspots and future development trends in this field. Oxidative stress refers to a physiological state in which the imbalance of the redox system leads to excessive accumulation of free radicals, which plays a vital role in the pathogenesis of NAFLD. Oxidative stress can improve membrane permeability by causing membrane phospholipid peroxidation, thus promoting the release of inflammatory factors, which act on Kupper cells, hepatic stellate cells and hepatocytes, and finally induce the progress of NAFLD (46). Studies have confirmed that some foods, dietary patterns and nutrients can effectively reduce the level of oxidative stress in the body to achieve the effect of treating NAFLD, such as fish, lean meat (47), nuts (48) and other foods; such as the Mediterranean diet (49–51), DASH (Dietary Approaches to Stop Hypertension) diet (52), RCR (Calorie-restricted regimen) diet (53) and other dietary patterns; such as carotenoids (54, 55), vitamin C (56), vitamin E (57), polyunsaturated fatty acids (58), methionine (59), isoflavones (60) and resveratrol (61). It is worth mentioning that Marinello et al. (62) found that organic acids in the diet can effectively treat NAFLD. Still, the combination of organic acids and ethanol will aggravate oxidative stress, which means that it is not feasible to treat alcoholic fatty liver with organic acids. Proper exercise can improve the level of antioxidant enzymes, enhance the ability of free radical scavenging, and play a role in preventing more serious oxidative stress damage. Csader et al. (63) proposed that 12 weeks of high-intensity intermittent exercise could change the expression of fat genes without changing the level of oxygen phospholipids. Cook et al. (64) found that restoring redox imbalance mediated by resistance exercise (the utilization of the pentose phosphate pathway) is very important for developing NAFLD. Fernandes et al. (65) found that aerobic exercise can protect against oxidative damage by improving non-enzymatic antioxidant defense but cannot change enzymatic defense. In addition, Barsalani (66) found that the effect of adding isoflavones in exercise training Is more evident than that provided by individual exercise.

Aerobic ability is the ability of the human body to obtain energy through aerobic metabolism when engaged in long-term and moderate-intensity exercise. Aerobic exercise can increase the oxidation function in the liver, reduce free fatty acids and reduce the synthesis of fat in the liver by improving aerobic endurance, thus treating NAFLD. Zhou (67) was included in the randomized controlled trial published in 2010–2021. The results showed that the best intervention effect on blood lipid and liver enzymes in NAFLD patients was aerobic training combined with resistance training followed by high-intensity interval training, resistance training, and aerobic training. Katsagon (68) was included in 20 randomized controlled trials published in 2005–2016. The results showed that resistance exercise was better than aerobic exercise in treating NAFLD. In addition, moderate to high-intensity and moderate-intensity continuous training is more beneficial than low-intensity continuous training and high-intensity interval training. Sabag (69) found that high-intensity interval training and moderate-intensity continuous training have the same effect on improving NAFLD. It can be seen that the impact of various aerobic exercise interventions on NAFLD is still uncertain, so further research is needed

## 5 Restrictions

There are some limitations in this research. First of all, all the data used in the analysis are only from the WOS core database, not all the databases of WOS. Secondly, the types of articles are limited to papers, and other influential articles published in other forms may also be ignored. In addition, WOS is constantly being updated dynamically; we may miss some new research progress. This has a particular influence on our results.

## 6 Conclusion

To sum up, this study makes a bibliometric analysis of the field of diet or exercise therapy for NAFLD from 2013 to 2023 for the first time, which helps us to deepen our understanding of this field and provides a valuable summary for discussing development trends and hot topics. Chinese scholars have contributed the most to this field, followed by scholars from the United States. The latest research results in this field can be found in *Nutrients*, *Frontiers in Nutrition*, and *Clinical Nutrition*. Professor Younossi Zobair M. and Professor Tur Josep A. were highly esteemed academic leaders in this field. In recent years, the research focus in this field has changed from “metabolic syndrome,” “population,” “steatohepatitis,” “alanine aminotransferase level,” “skeletal muscle,” “cardiovascular risk,” “coronary heart disease,” and “activated protein kinase” to “cross-sectional study,” “oxidative stress” and “aerobic capacity.”

## Author contributions

XS: Writing – original draft. YX: Writing – original draft. HS: Writing – original draft. FR: Writing – original draft. NT: Writing – original draft. LZhu: Writing – original draft. SL: Writing – original draft. JW: Writing – review & editing. LZha: Writing – review & editing. SY: Writing – review & editing. GJ: Writing – review & editing. BL: Writing – review & editing. NW: Writing – review & editing.
